# The Association Between Lymphovascular or Perineural Invasion in Radical Prostatectomy Specimen and Biochemical Recurrence

**DOI:** 10.3390/cancers16213648

**Published:** 2024-10-29

**Authors:** Carolin Siech, Mike Wenzel, Nico Grosshans, Cristina Cano Garcia, Clara Humke, Florestan Johannes Koll, Zhe Tian, Pierre I. Karakiewicz, Luis A. Kluth, Felix K. H. Chun, Benedikt Hoeh, Philipp Mandel

**Affiliations:** 1 Goethe University Frankfurt, University Hospital, Department of Urology, 60590 Frankfurt am Main, Germany; 2 Cancer Prognostics and Health Outcomes Unit, Division of Urology, University of Montréal Health Center, Montréal, QC H2X 3E4, Canada

**Keywords:** BCR, lymphovascular invasion, perineural invasion, radical prostatectomy, prostate cancer

## Abstract

Objective: The aim of this study was to test for the association between lymphovascular invasion or perineural invasion in radical prostatectomy (RP) specimens and biochemical recurrence (BCR). Methods: Relying on a tertiary-care database, we identified prostate cancer patients treated with RP between January 2014 and June 2023. Of these, the majority underwent robotic-assisted RP (81%). Kaplan–Meier survival analyses and Cox regression models addressed BCR according to either lymphovascular invasion or perineural invasion in RP specimens. Additionally, the linear trend test assessed the association between the Gleason Grade Group or pathologic tumor stage and lymphovascular or perineural invasion. Results: Of 822 patients, 78 (9%) exhibited lymphovascular invasion and 633 (77%) exhibited perineural invasion in RP specimens. In survival analyses, the five-year BCR-free survival rates were 62% in patients with lymphovascular invasion vs. 70% in patients without lymphovascular invasion (*p* = 0.04) and 64% in patients with perineural invasion vs. 82% in patients without perineural invasion (*p* = 0.01). In univariable Cox regression models, lymphovascular invasion (hazard ratio 1.58, 95% confidence interval 1.01–2.47; *p* = 0.045) and perineural invasion (hazard ratio 1.77, 95% confidence interval 1.13–2.77; *p* = 0.013) were both associated with a higher BCR rate. After accounting for age at surgery, PSA value, pathologic tumor stage, Gleason Grade Group, lymph node invasion, positive surgical margin, surgical approach, and adjuvant radiation therapy, lymphovascular (*p* = 0.740) or perineural invasion (*p* = 0.341) were not significantly associated with a higher BCR since the Gleason Grade Group and pathologic tumor stage highly correlated with lymphovascular as well as perineural invasion. Conclusions: In univariable models, lymphovascular or perineural invasion is associated with BCR. After adjustment for standard pathologic tumor characteristics, lymphovascular or perineural invasion is not an independent predictor for BCR.

## Simple Summary

The prognostic value of lymphovascular or perineural invasion in prostate cancer specimens regarding oncological outcomes after radical prostatectomy is unclear. Within a contemporary study cohort of 822 prostate cancer patients, 78 (9%) exhibited lymphovascular invasion and 633 (77%) exhibited perineural invasion in RP specimens. In univariable Cox regression models, lymphovascular invasion and perineural invasion were both associated with higher rates of biochemical recurrence (BCR). However, after multivariable adjustment for standard pathologic tumor characteristics, lymphovascular or perineural invasion was not found to be an independent predictor for BCR. These phenomes may be explained by the strong association between the Gleason Grade Group and pathologic tumor stage with lymphovascular as well as perineural invasion.

## 1. Introduction

In patients with localized prostate cancer (PCa), radical prostatectomy (RP) has emerged as a standard of care [1,2,3,4]. Despite curatively intended therapy, some patients may develop biochemical recurrence (BCR) in the postoperative course [5,6,7]. Prognostic factors for BCR of localized PCa after RP include clinical factors such as age [8] and prostate specific antigen (PSA) [9]. In addition, several pathologic features in RP specimens are associated with worse short- and long-term oncological outcomes, such as non-organ confined pathologic tumor stage [10,11], Gleason Grade Group ≥4 [10,12,13], and positive surgical margins [5,7]. However, the clinical impact as well as the prognostic value of lymphovascular invasion in PCa specimens regarding oncological outcomes, such as biochemical recurrence after RP, remain unclear [14,15,16,17,18,19,20,21]. Moreover, while the prognostic role of perineural invasion in prostate cancer samples collected by needle core biopsy is being currently discussed [22,23,24], the prognostic role of perineural invasion in contemporary RP specimens remains controversial [20,25,26,27,28].

We addressed this uncertainty and hypothesized that BCR rates after RP are higher in patients with lymphovascular invasion or perineural invasion compared to those without the respective pathologic feature in RP specimens. To address this hypothesis, we relied on a contemporary cohort of PCa patients treated with RP in a tertiary care referral center.

## 2. Materials and Methods

### 2.1. Study Population

**Figure 1:**
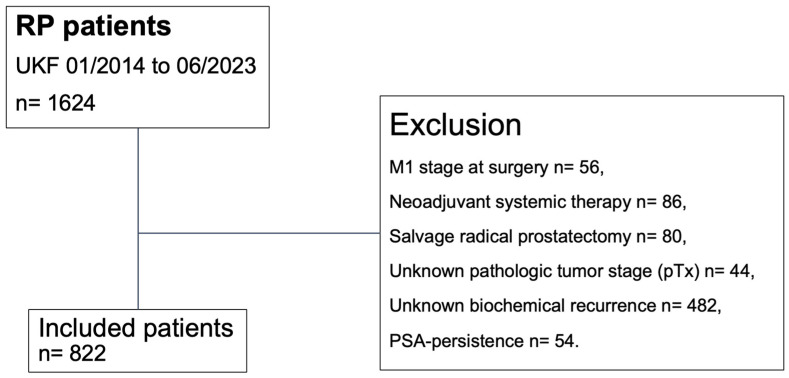
Consort diagram. Abbreviations: PSA= prostate-specific antigen; pT = pathologic tumor stage at surgery; RP = radical prostatectomy; UKF = University Hospital Frankfurt.

Using our prospectively maintained institutional tertiary-care database, we retrospectively identified patients with histologically confirmed adenocarcinoma of the prostate who underwent RP between January 2014 and June 2023 at the Department of Urology of the Goethe University Hospital Frankfurt, Germany (Figure 1) [29]. In the current study, only patients with known follow-up records of BCR were included. Patients with persistent PSA, defined as post-RP PSA of >0.1 ng/mL within six weeks after surgery, were excluded from the study cohort [30]. Further exclusion criteria consisted of clinical suspicion of metastases at time of surgery (cM1), treatment with neoadjuvant systemic therapy (chemotherapy and/or hormonal therapy), and previous radiation therapy of the prostate (salvage RP). Moreover, patients with an unknown pathologic tumor stage (pTx) were excluded. Informed written consent to participate in this study was given by all patients. Approval by the local ethics committee was obtained prior to data collection. All reporting was reviewed in accordance with the precepts established by the Declaration of Helsinki.

### 2.2. Definition of Variables for Analyses

BCR, the primary endpoint of the study, was derived from patients’ self-reports in follow-up and was defined according to American Urological Association (AUA) guidelines as an initial serum PSA value of ≥0.2 ng/mL, with a second confirmatory level of >0.2 ng/mL in follow-up after RP [6,30,31]. Lymphovascular invasion as well as perineural invasion were determined by specialized uropathologists. All pathologic diagnoses were confirmed by a second pathologist.

Covariates consisted of age at surgery (continuously coded), PSA value (continuously coded), pathologic tumour stage (pT2 vs. pT3/pT4), Gleason Grade Group (1 vs. 2 vs. 3 vs. 4 vs. 5), pathologic lymph node stage (pN0 vs. pN1 vs. pNx), positive surgical margin (no vs. yes vs. unknown), surgical approach (open vs. robotic-assisted), and adjuvant radiation therapy (no vs. yes).

### 2.3. Statistical Analyses

Four analytical steps were performed. First, baseline characteristics were tabulated. Descriptive statistics included medians and interquartile ranges (IQRs) for continuously coded variables and frequencies and proportions for categorical variables. Second, Kaplan–Meier survival analyses addressed BCR-free survival according to either lymphovascular or perineural invasion. Third, univariable, and multivariable Cox regression models addressed BCR. Moreover, we relied on testing for proportional hazards assumption for a Cox regression model fit [32]. Additionally, linear trend tests assessed the association between the Gleason Grade Group or pathologic tumor stage and lymphovascular or perineural invasion.

The R software environment was used for statistical computing and graphics (R version 4.2.2; R Foundation for Statistical Computing, Vienna, Austria) [33]. All tests were two sided, with a level of significance set at *p* < 0.05.

## 3. Results

### 3.1. Descriptive Characteristics

Relying on our institutional tertiary-care database of 1624 PCa patients who underwent RP between January 2014 and June 2023, we identified 822 patients according to the inclusion criteria (Table 1). Overall, the median follow-up of the study cohort was 20 months (IQR: 10–38). Of those, 78 (9%) exhibited lymphovascular invasion and 633 (77%) exhibited perineural invasion in RP specimens.

### 3.2. Biochemical Recurrence Rates According to Lymphovascular Invasion

**Figure 2:**
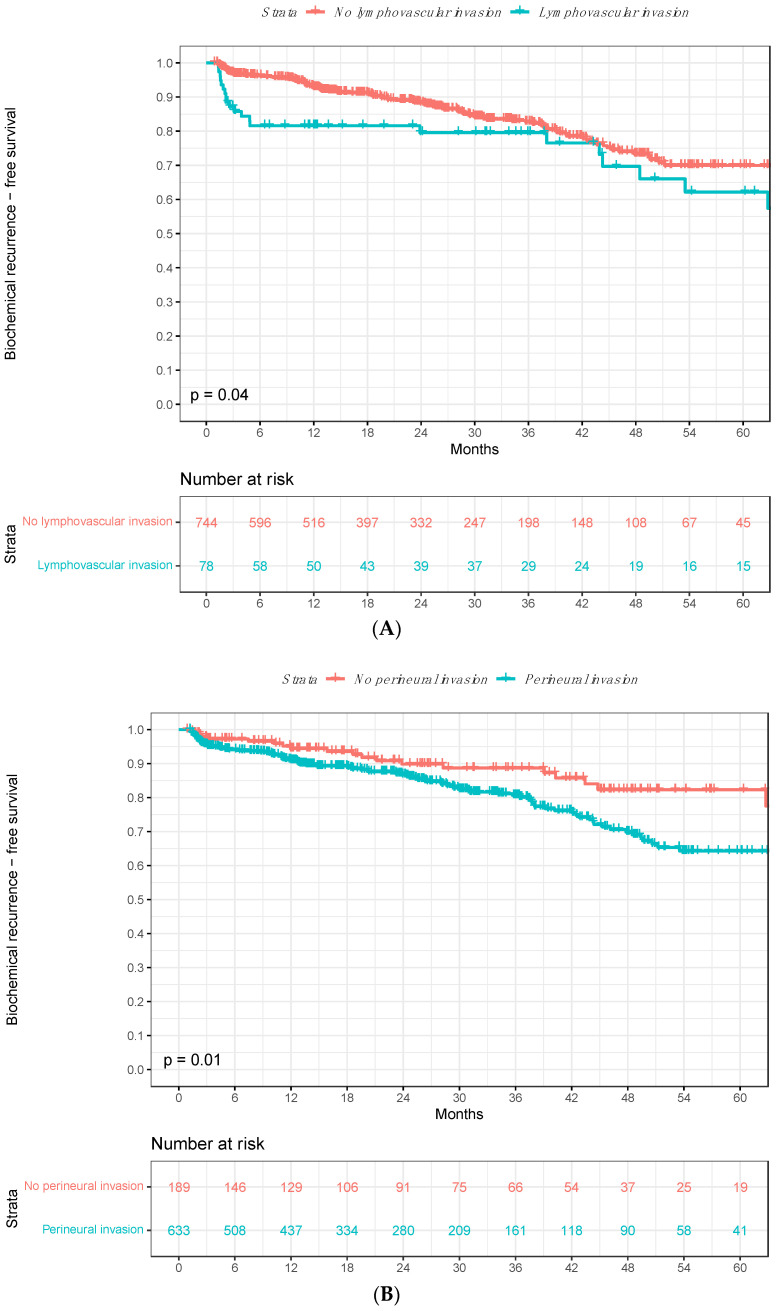
Kaplan–Meier survival analyses addressing biochemical recurrence (BCR)-free survival after radical prostatectomy (RP) according to presence vs. absence of (**A**) lymphovascular invasion and (**B**) perineural invasion in RP specimens. Abbreviations: BCR = biochemical recurrence; BCRFS = biochemical recurrence-free survival; RP = radical prostatectomy.

**Figure 3:**
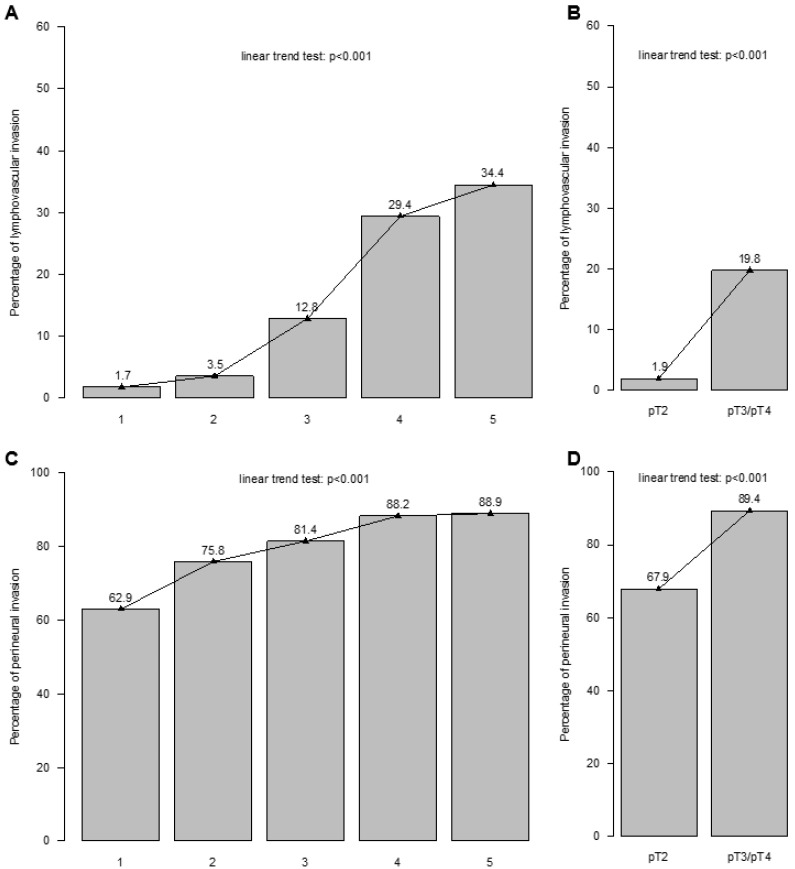
The association between (**A**) Gleason Grade Group and lymphovascular invasion, (**B**) pathologic tumor stage (pTstage) and lymphovascular invasion, (**C**) Gleason Grade Group and perineural invasion, and (**D**) pTstage and perineural invasion in radical prostatectomy specimen. Abbreviation: pT = pathologic tumor stage.

The five-year BCR-free survival rates were 62% in patients with lymphovascular invasion vs. 70% in patients without lymphovascular invasion in Kaplan–Meier survival analyses (Δ8%; *p* = 0.04; Figure 2A). In univariable Cox regression models, lymphovascular invasion predicted higher BCR rates after RP (hazard ratio [HR]: 1.58, 95% confidence interval [CI]: 1.01–2.47; *p* = 0.045; Table 2). After adjustment for age at surgery, PSA value, pathologic tumor stage, Gleason Grade Group, lymph node invasion, positive surgical margin, surgical approach, and adjuvant radiation therapy in multivariable models, lymphovascular invasion was not significantly associated with higher BCR rates (HR 0.91, 95% CI 0.50–1.63; *p* = 0.740) since the Gleason Grade Group and pathologic tumor stage highly correlated with higher rates of lymphovascular invasion in the linear trend test (*p* < 0.001; Figure 3A,B).

### 3.3. Biochemical Recurrence Rates According to Perineural Invasion

In Kaplan–Meier survival analyses, the five-year BCR-free survival rates were 64% in patients with perineural invasion vs. 82% in patients without perineural invasion (Δ18%; *p* = 0.01; Figure 2B). In univariable Cox regression models, perineural invasion was statistically significantly associated with BCR after RP (HR 1.77, 95% CI 1.13–2.77; *p* = 0.013; Table 2). After adjustment for age at surgery, PSA value, pathologic tumor stage, Gleason Grade Group, lymph node invasion, positive surgical margin, surgical approach, and adjuvant radiation therapy in multivariable models, perineural invasion was not significantly associated with higher BCR rates (HR 1.26, 95% CI 0.78–2.04; *p* = 0.341) since the Gleason Grade Group and pathologic tumor stage highly correlated with higher rates of perineural invasion in the linear trend test (*p* < 0.001; Figure 3C,D).

## 4. Discussion

We hypothesized that BCR rates after RP are higher in patients with lymphovascular invasion or perineural invasion compared to those without the respective pathologic feature in RP specimens. Addressing this hypothesis in a contemporary cohort of PCa patients treated with RP at a tertiary care referral center between January 2014 and June 2023, we made several important observations.

First, we tabulated the proportion of patients diagnosed with lymphovascular invasion in our contemporary cohort of localized PCa patients treated with RP. Specifically, 78 of 822 patients (9%) exhibited lymphovascular invasion in RP specimens. Lymphovascular invasion rates in RP specimens in the literature range from 10 to 30% [14,15,16,20]. Thus, the lymphovascular invasion rate of 9% reported in the present study is slightly lower. However, these small differences in lymphovascular invasion rates may be explained by differences in pathologic tumor characteristics (e.g., pathologic tumor stage, Gleason Grade Group) in the study cohort [16,20,34]. As a consequence, to reduce uncontrolled bias or confounding due to differences in pathologic tumor characteristics, it is essential to adjust for these covariates in multivariable models, as in the present study.

Second, we recorded the proportion of patients diagnosed with perineural invasion in our contemporary cohort of localized PCa patients treated with RP. Specifically, 633 of 822 patients (77%) exhibited perineural invasion in RP specimens. The currently reported perineural invasion rate in RP specimens demonstrates that perineural invasion represents a common pathologic feature in RP specimens. Moreover, it is consistent with those reported in previous historical series, ranging from 44 to 78% [20,25,26,27,28,35]. This broad variability observed across different studies may be explained by differences in patient populations as well as interobserver variability. Therefore, it is essential to rely on a central pathology review of RP specimens, as in our tertiary referral center.

Third, we identified a strong association between the Gleason Grade Group and pathologic tumor stage with lymphovascular and perineural invasion in RP specimens in the linear trend tests. The lymphovascular invasion rate increased from 1.7% in Gleason Grade Group 1 to 34.4% in Gleason Grade Group 5 and from 1.9% in pT2 to 19.8% in pT3/pT4. This observation is not unexpected since lymphovascular invasion, defined as the invasion of vessel walls by tumor cells and/or the presence of tumor emboli surrounded by endothelial cells, represents a pathologic tumor feature that occurs in an advanced stage of PCa [34]. Similarly, the perineural invasion rate increased from 62.9% in Gleason Grade Group 1 to 88.9% in Gleason Grade Group 5 and from 67.9% in pT2 to 89.4% in pT3/pT4. To the best of our knowledge, we are the first to graphically depict these associations.

Fourth, in survival analyses, we identified important differences in BCR after RP according to the presence vs. absence of lymphovascular invasion in RP specimens. The five-year BCR-free survival rates were 62% in patients with lymphovascular invasion vs. 70% in patients without lymphovascular invasion (Δ8%; *p* = 0.04). Similarly, lymphovascular invasion predicted a 1.6-fold higher BCR rate after RP in univariable Cox regression models (*p* = 0.045). However, in multivariable models adjusting for age at surgery, PSA value and other pathologic tumor characteristics, including pathologic tumor stage, Gleason Grade Group, lymph node stage, positive surgical margin, surgical approach and adjuvant radiation therapy, the BCR rate did not statistically significantly differ according to the presence vs. absence of lymphovascular invasion in RP specimens (*p* = 0.740). The lack of consistent association of lymphovascular invasion in RP specimens with BCR may be explained in several ways. First, it may be postulated that the lack of association between lymphovascular invasion and BCR after multivariable adjustment for pathologic tumor characteristics and adjuvant radiation therapy relates to a stronger association between the Gleason Grade Group as well as pathologic tumor stage and lymphovascular invasion in RP specimens, as described above. Second, lymphovascular invasion is associated with a lower prognostic strength in the current study cohort compared to historical reports [14,20]. Third, recently published analyses by Kawase et al. indicate that lymphovascular invasion may be of prognostic value only in select RP patients [15]. Moreover, other unmeasured variables may underly this lack of association. For example, the magnitude of differences in BCR rates between patients with vs. without lymphovascular invasion may be too small to detect independent predictor status. The proposed explanations are preliminary at best. Nevertheless, contemporary prostate cancer guidelines of the European Association of Urology (EAU) recommend reporting lymphovascular invasion as a mandatory element in pathology reports [30]. Moreover, the presence of lymphovascular invasion appears to be associated with pathogenic germline DNA-repair gene mutations in men with PCa [36].

Finally, we also addressed BCR after RP according to the presence vs. absence of perineural invasion in RP specimens. In Kaplan–Meier survival analyses, five-year BCR-free survival rates were 64% in patients with perineural invasion vs. 82% in patients without perineural invasion (Δ18%; *p* = 0.01). In univariable Cox regression models, perineural invasion predicted a 1.8-fold higher BCR rate after RP (*p* = 0.013). However, perineural invasion was not statistically significantly associated with BCR after RP in multivariable Cox regression models (*p* = 0.341). These observations validate historical studies, where perineural invasion did not provide additional information for BCR risk prediction after accounting for standard pathologic tumor characteristics, such as pathologic tumor stage, Gleason score, and positive surgical margin [25,27,37]. However, these findings are in contrast to observations recorded by Kang et al. and Stankovic et al., who identified perineural invasion as an independent predictor of BCR after RP [20,26]. Nonetheless, analyses by Wu et al. suggest that a minimum of three foci of perineural invasion is required to predict BCR [27]. Moreover, Gertsen et al. recently reported that apex-localized perineural invasion independently predicted higher BCR compared to mid- or base-localized perineural invasion [38]. In consequence, analyses categorizing perineural invasion as presence vs. absence may underestimate the prognostic value of perineural invasion in RP specimens.

Taken together, in our contemporary study cohort, only 9% of patients exhibited lymphovascular invasion and 77% exhibited perineural invasion in RP specimens. In univariable models, lymphovascular or perineural invasion are both associated with BCR. However, after adjustment for age at surgery, PSA value, standard pathologic tumor characteristics, surgical approach, and adjuvant radiation therapy, lymphovascular or perineural invasion were not statistically significantly associated with BCR. Nevertheless, our observations may indicate that reporting lymphovascular or perineural invasion in RP specimens may not provide additional prognostic value beyond standard pathologic tumor characteristics.

Despite our important observations, the present study has limitations. First, due to its retrospective nature, a potential for residual selection biases, despite systematic adjustment for biases and confounders in multivariable models, remained. This limitation is applicable to all studies relying on a retrospective study design [14,20,25,34]. Second, our study relies on a limited sample size. Specifically, only 24 patients with lymphovascular invasion and 115 patients with perineural invasion experienced BCR. In consequence, further sub-stratification such as stage-specific subgroup analyses was not possible. Third, we relied on a study cohort of PCa patients treated with RP between 2014 and 2023. The study period of around 10 years may introduce biases, as medical treatment protocols as well as surgical techniques have slightly changed over time. However, this limitation applies to both patients with vs. without lymphovascular or perineural invasion in RP specimens. Fourth, in our cohort, lymphovascular invasion as well as perineural invasion were categorized as presence vs. absence. The determination of the vascular status in RP specimens must be evaluated with great caution in the event of negative findings. Unlike in other organs, a more intensive immunohistochemical examination would be required for a more reliable assessment. Nonetheless, previous analyses indicate that ambiguous RP specimens exhibit similar prognostic outcomes compared to those classified as positive [39]. Moreover, the extent of perineural and vascular invasion as well as their localization (intra- vs. extratumoral) should be determined in future studies [27,28]. Finally, postoperative follow-up within our study cohort was also limited. Therefore, other study endpoints that could be equally as interesting as BCR, such as metastasis-free, cancer-specific or overall survival, could not be addressed.

## 5. Conclusions

In univariable models, lymphovascular or perineural invasion is associated with BCR. However, after adjustment for standard pathologic tumor characteristics, lymphovascular or perineural invasion is not an independent predictor for BCR.

## Figures and Tables

**Table 1:** Descriptive characteristics of 822 prostate cancer patients treated with radical prostatectomy (RP) between January 2014 and June 2023.

Characteristic		Overall *n* = 822 ^1^
Age at surgery (in years)		66 (61, 71)
PSA (in ng/mL)		7.1 (5.2, 10.5)
pTstage	pT2	473 (58%)
	pT3/pT4	349 (42%)
pNstage	pN0	692 (84%)
	pN1	42 (5%)
	pNx	88 (11%)
Gleason Grade Group	1	116 (14%)
	2	426 (52%)
	3	156 (19%)
	4	34 (4%)
	5	90 (11%)
Lymphovascular invasion		78 (9%)
Perineural invasion		633 (77%)
Positive surgical margin	no	565 (69%)
	yes	236 (29%)
	unknown	21 (2%)
Robotic-assisted radical prostatectomy		664 (81%)
Adjuvant radiation therapy		77 (10%)

^1^ Median (interquartile range); n (%). Abbreviations: pN = pathologic lymph node stage; PSA = prostate-specific antigen; pT = pathologic tumor stage.

**Table 2:** Univariable and multivariable Cox regression models addressing rates of biochemical recurrence (BCR) after radical prostatectomy (RP), according to presence vs. absence of lymphovascular invasion or perineural invasion in RP specimens.

	Univariable			Multivariable *		
	HR	95% CI	*p*-Value	HR	95% CI	*p*-Value
Lymphovascular invasion (Ref. no)	**1.58**	1.01, 2.47	**0.045**	0.91	0.50, 1.63	0.740
Perineural invasion (Ref. no)	**1.77**	1.13, 2.77	**0.013**	1.26	0.78, 2.04	0.341

* adjusted for age at surgery, PSA value, pTstage, Gleason Grade Group, pNstage, positive surgical margin, surgical approach, and adjuvant radiation therapy. Abbreviations: CI = confidence interval; HR = hazard ratio; pN = pathologic lymph node stage; PSA = prostate-specific antigen; pT = pathologic tumor stage. Bold highlights statistically significant hazard ratio and corresponding *p*-value.

## Data Availability

All data generated or analyzed during this study are included in this article. Further enquiries can be directed to the corresponding author.
